# Categories, applications, and potential of stem cells in bone regeneration: an overview

**DOI:** 10.3389/fmed.2025.1606100

**Published:** 2025-08-20

**Authors:** Mingyang Jiao, Ting Shuai, Zhongfang Zhao, Yuwei Wu, Linwei Yu, Jingwen Sun, Raffaele De Caro, Veronica Macchi, Andrea Porzionato, Elena Stocco, Chanyuan Jin

**Affiliations:** ^1^Beijing Key Laboratory of Digital Stomatology, NHC Key Laboratory of Digital Stomatology, NMPA Key Laboratory for Dental Materials, Department of Prosthodontics, National Center for Stomatology, National Clinical Research Center for Oral Diseases, National Engineering Research Center of Oral Biomaterials and Digital Medical Devices, Peking University School and Hospital of Stomatology, Beijing, China; ^2^The Second Clinical Division of Peking University School and Hospital of Stomatology, Beijing, China; ^3^Hospital of Stomatology, Lanzhou University, Lanzhou, China; ^4^School of Acupuncture-Moxibustion and Tuina, Beijing University of Chinese Medicine, Beijing, China; ^5^Section of Human Anatomy, Department of Neuroscience, University of Padova, Padua, Italy; ^6^Department of Women’s and Children’s Health, University of Padova, Padua, Italy; ^7^Department of Surgery, Oncology and Gastroenterology, University of Padova, Padua, Italy

**Keywords:** stem cells, bone tissue engineering, biomaterials, bone regeneration, regenerative medicine, osteogenic differentiation

## Abstract

Bone defects affect many individuals globally and can result in significant suffering and impairment, particularly among the elderly population. In addition, current treatment options for critical-size bone defects, such as autologous or allogeneic bone graft transplantation, present significant challenges. Within this clinical scenario the identification of novel and effective approaches for bone regeneration is urgently needed, and options derived from tissue engineering may be particularly appealing. Bone tissue engineering for bone regeneration involves the application of seed cells, growth factors, and biomaterials to create bioactive substitutes for repairing bone defects. In recent decades, advancements in stem cell research and biological biomaterials have led to remarkable breakthroughs in the field of bone regeneration. In particular, various categories of stem cells have been isolated, characterized, and employed in tissue engineering approaches. This review summarizes the applications of the main types of stem cells currently used for bone regeneration through tissue engineering approaches, and it also pays attention to the most appealing materials for it.

## 1 Introduction

Approximately 15% of the whole-body weight consists of hard bone. Of this, cortical bone (the outer layer) accounts for about 80% of total adult bone mass. It has a relatively low porosity of 3%–5%, it is highly resistant to mechanical loads (bending and torsion) and it can sustain weight along with providing structural integrity and physical support (1). The remaining ∼ 20% of adult bone mass is made up of the cancellous bone (the inner layer), which is characterized by a honeycomb-like trabecular connection and a porosity of about 80%–90% (1). Besides, the bone is composed of organic components, that include type I collagen, non-collagenous proteins and inorganic components (i.e., hydroxyapatite crystals formed from calcium and phosphate ions). The interaction of these elements determines bone’s mechanical properties, including compressive strength and fracture toughness (2–4). Furthermore, along with its supporting function, bone plays a role in muscular function, hematopoiesis, and protection of internal organs including that of the nervous system (1). Considering the fundamental role of bone within the body, this tissue is extensively studied and bone defects, resulting from several conditions (trauma, cancer, infection, surgical complication, osteoporosis), are a global concern significantly compromising life quality (5–8). While bone distinguishes for ability to self-regenerate in case of minor injuries, defects that are 1.5 times the diameter of the bone are considered as critical-sized defects, requiring surgery to avoid non-union, malunion, or pathological fractures (1, 9).

The autologous or allogeneic bone graft transplantation (known as bone grafting) is currently considered the gold standard approach for treating bone defects (10). Autologous bone grafting involves using bone tissue from the patient’s own distal donor site. This approach does not induce immune rejection and the implants possess osteoinductive capacity. However, obtaining bone grafts is often faced with the challenges of limited tissue availability or various postoperative consequences, including enduring pain with possible sensory loss, infection, bleeding at the donor site, prolonged wound drainage, need for second surgery; consequently, this strategy is not adequate for children or aging patients (1, 11). Allografts are decellularized matrices taken from donor patients, displaying a bone structure and extracellular matrix (ECM) comparable to the original bone. Differently from autografts, allografts undergo lower integration and vascularization in the implant site. Additionally, they present the risk of spreading infections or triggering an immunological rejection; despite cryogenic treatments that may reduce immune rejection, mechanical strength may be correspondingly affected. Compared to autografts, allografts also display a reduced osteoinductive behavior and no cellular component, due to irradiation or freeze-drying processing they are exposed to (12). It is rare for either of these methods to fully restore the full function of injured bone tissue (13–15); however, autologous bone grafts are still considered the gold standard for bone defects management (11). Thus, considering these significant limits, researchers dedicated toward the identification of alternative on-the-bench smart substitutes. Since 1987, merging materials science and cell biology skills, a new field called “Tissue Engineering” stood out (16). The aim of this field is to address the scarcity of tissues/organs accessible for transplantation. In the case of bone, the goal is to mitigate the drawbacks associated with the aforementioned treatment options, including donor site injury, infection transmission, immunological rejection, and limited availability (12), while enhancing patient prognosis by substituting damaged tissue with a comparable one in terms of structure and function (2). This process involves utilizing scaffolds, seed cells, and biologically active growth factors to create bioactive substitutes that can restore normal bone function and repair areas of bone defects.

Together with an increasing understanding of material science, advancements in molecular biology, cell biology, and biochemistry have played a significant role in enhancing our comprehension of cell differentiation mechanisms and tissue growth, along with the cell-ECM interactions. Consequently, the use of stem cells has gained significance in the process of bone regeneration via tissue engineering: together with a great ability to proliferate, they also show a reduced inclination toward senescence versus differentiated cells. Additionally, several studies have demonstrated that stem cells can induce osteogenesis, thus resulting beneficial in the treatment of profound bone defects as consequence of trauma or inadequate blood supply (17, 18).

This narrative review focuses on the major categories of stem cells in bone tissue engineering and considers the main advancements on their use in this field. In addition, biomaterials employed in bone tissue engineering are presented as an extensive representation of this appealing and promising method of regeneration.

## 2 Different sources of stem cells for bone regeneration

Stem cells, that can be recognized both in embryos and in adult tissues, are defined as unspecialized cells endowed with the ability to self-renew and to differentiate into more than one cell lineage. Typically, there are different steps of specialization that include (19–21): totipotent stem cells, pluripotent stem cells, multipotent stem cells, oligopotent stem cells and unipotent stem cells. Totipotent stem cells (e.g., zygote, up to 4 days from egg fertilization) have the highest differentiation potential; they can form both embryo and extra-embryonic structures and can divide and differentiate into cells of the whole organism. Pluripotent stem cells (e.g., embryonic stem cells, inner cell mass of pre-implantation embryos) form cells of all germ layers except for extraembryonic structures. Multipotent stem cells (e.g., hematopoietic stem cells) can specialize in discrete cells of specific cell lineages; oligopotent stem cells (e.g., myeloid stem cells) can differentiate into several cell types. Unipotent stem cells have the narrowest differentiation capabilities and can divide repeatedly (19). Generally, these cells can differentiate into specialized tissue when exposed to a suitable environment (22).

**FIGURE 1:**
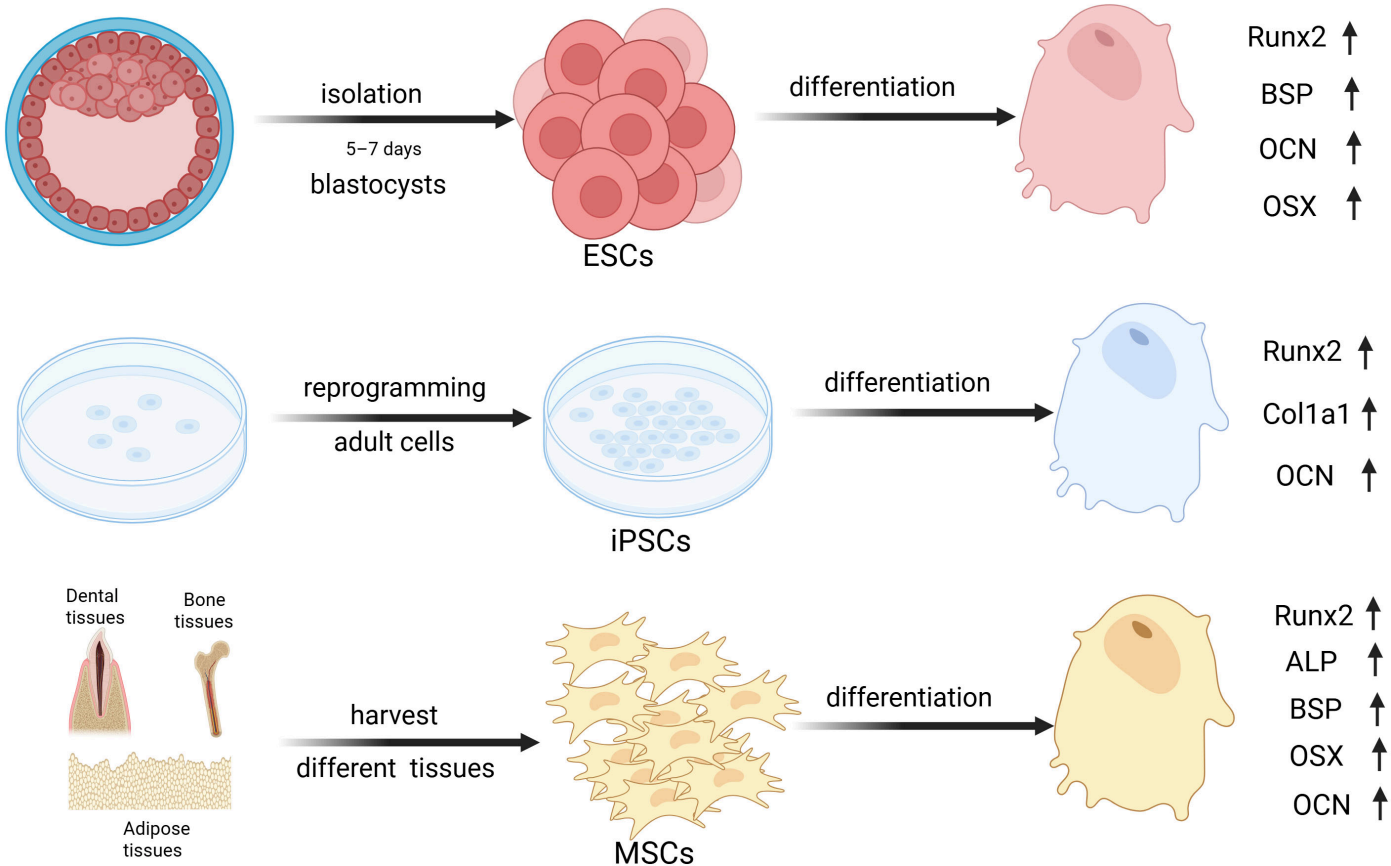
The graphs of osteogenic markers across An error in the conversion from LaTeX to XML has occurred here. 18 different stem cell types. Runx2, Runt-related transcription factor 2; BSP, Bone sialoprotein; OCN, Osteocalcin; OSX, Osterix; Col1a1, Collagen Type I Alpha 1 Chain; ALP, Alkaline phosphatase. Created with BioRender.com.

The identification of suitable cells for bone regeneration encompasses many factors. Together with the ability to differentiate into osteoblasts, it is important to assess whether the cells can be easily accessed, the quantity that can be obtained and whether the source of the cells may raise ethical concerns (23). This section will specifically discuss three types of stem cells: mesenchymal stromal cells (MSCs), embryonic stem cells, and induced pluripotent stem cells. Currently, MSCs are the most studied type, embryonic stem cells and induced pluripotent stem cells are gaining popularity among researchers despite issues (mainly related to safeness and ethics) for future translation in clinical practice. Figure 1 is a schematic diagram of the acquisition methods along with osteogenic differentiation markers of these three types of cells.

### 2.1 Mesenchymal stromal cells: therapeutic promise and the challenge of senescence

Mesenchymal stromal cells are a multipotent and heterogeneous cell population with the ability to differentiate into numerous types including chondrocytes, myoblasts, osteoblasts, and adipocytes (24). According to the International Society for Cellular Therapy (ISCT), there are three widely accepted criteria to define MSCS; they must: (i) be plastic-adherent in standard culture conditions; (ii) show a multipotent phenotype (i.e., ability to differentiate into adipocytes, osteoblasts and chondrocytes); (iii) exhibit the presence of CD73, CD90, and CD105 surface markers but not CD11b, CD14, CD19, CD34, CD45, CD79α, and the HLA-DR (25). There are several MSCs sources within the body (bone marrow, adipose tissue, periosteum, vessels wall, peripheral circulation, muscle, tendon, umbilical cord blood, skin, dental tissues). For clinical applications such as fracture repair, an ideal MSC source should be easily accessible, harvestable through non-invasive procedures, capable of rapid *in vitro* expansion, and able to survive and integrate effectively at the implantation site without promoting tumorigenesis (26).

However, a major limitation in using autologous MSCs for therapeutic purposes relies on the phenomenon of cellular senescence, which significantly compromises the cells regenerative potential. Typically, senescent MSCs distinguish for an enlarged and flattened aspect, presence of cytoplasmatic granuli and increased lysosomal mass (27). All these characteristics are well identifiable during long-term culture *in vitro* and are associated with reduced proliferation, lower adhesion to plastic surface, impaired colony-forming ability, and a marked decline in osteogenic differentiation potential (28, 29). Moreover, senescent MSCs often tend to differentiate toward adipose tissue (30, 31). While the MSCs characterizing surface markers (CD73, CD90, CD105) remain stable with senescence, others are downregulated (CD106 and CD146) or upregulated (CD264 and CD295) (31–33). Biomarkers like senescence-associated β-galactosidase (SA-β-gal) and α-L-fucosidase (SA-α-Fuc) are commonly employed to identify senescent cells (27, 34). At the molecular level, senescence is associated with irreversible cell cycle arrest mediated by the upregulation of p53/p21WAF1/CIP1 and p16INK4A (35). Additionally, senescent MSCs develop a senescence-associated secretory phenotype (SASP) with secretion of pro-inflammatory cytokines (e.g., IL-6, IL-8), growth factors, and proteases. The SASP factors together with perpetuating senescence also induce premature senescence in neighboring cells via paracrine signaling, ultimately disrupting tissue regeneration and immune modulation (36, 37).

Though MSCs are widely recognized for their immunosuppressive and anti-inflammatory roles, senescent MSCs can exhibit pro-inflammatory behavior, contributing to tissue dysfunction and impaired healing (38, 39). Considering that MSCs potential for proliferation/differentiation decreases with patient’s age and age-associated comorbidities, allogeneic MSCs can be used instead of autologous MSCs, preserving regenerative efficacy (19, 40).

Senescence in MSCs can arise from several mechanisms, including replicative senescence (due to telomere shortening), oncogene-induced senescence, stress-induced senescence, and developmental senescence. Interestingly, it seems that MSCs senescence is a modifiable risk factor, and intense research efforts are dedicated toward the identification of effective strategies to reverse senescence-associated changes allowing to enhance MSCs use as therapy as well as application for regenerative medicine purposes (34, 41).

To date, bone marrow and adipose-derived MSCs are the two most investigated types of stem cells. Furtherly, dental-derived types are becoming a desirable option for bone tissue engineering too (Table 1).

**TABLE 1:** Source, advantages, and disadvantages of different types of stem cells for bone regeneration.

Stem cell type	Source	Advantages	Disadvantages
BMMSCs	Bone marrow derived from long bone or jawbone during transplants or orthopedic surgeries	1. Accessible source for cell harvesting 2. Easy to cultivate *in vitro* 3. Ease of preparation 4. Can be easily induced to differentiate into osteoblasts	1. Painful acquisition process 2. Potential for more serious complications 3. Low content in bone marrow tissue 4. Risk of infection in bone tissue 5. Difficulties in obtaining sufficient quantity
ADSCs	Collection of superficial subcutaneous adipose tissue during liposuction or reconstructive surgery	1. Easy to obtain a sufficient quantity 2. Can be painless for patients to provide enough quantity 3. Can be cultured for a long time *in vitro* with a low apoptosis rate 4. Lower risk of infection 5. The osteogenic capacity does not decrease with cell proliferation	1. The freezing and subsequent thawing can reduce their ability 2. Cell contamination can reduce the proliferation and differentiation ability 3. Lower osteogenic capacity than BMMSCs 4. High tendency to differentiate into adipocytes
DMSCs	1. Dental pulp from exfoliated deciduous teeth or extracted permanent tooth 2. Periodontal or gingival tissues 3. Dental follicle tissues.	1. Can be easily harvested from medical waste 2. Can be relatively easy to access and less invasive 3. Higher proliferation rate	Lower osteogenic capacity
ESCs	Pre-implantation stage blastocyst’s inner cell mass after fertilization for 5–7 days	1. Can differentiate into any type of cell in the body 2. Have unlimited self-renewal capacity 3. Use as disease model	1. Destruction of an embryo at the blastocyst stage with consequent ethical issues 2. Are available only in limited quantities 3. Limited by the ethics and laws of many countries 4. High rate of mutation 5. Risk of causing teratoma
IPSCs	Several differentiated mature somatic cells	1. No ethical issues compared to embryonic stem cells 2. Easy to obtain 3. HLA histocompatibility 4. Use as disease model 5. Have the ability to be reprogrammed 6. Have a wider range of applications	1. Risk of tumor formation 2. Risk of genomic instability 3. Risk of immune rejection 4. Cost

#### 2.1.1 Bone marrow mesenchymal stromal cells

Bone marrow mesenchymal stromal cells (BMMSCs) were the earliest MSCs successfully separated and recognized as effective for bone tissue engineering. Together with the capacity for self-renewal, they also show multidirectional differentiation toward the osteoblast and chondrogenic lineages upon exposure to specific stimuli. Due to their advantageous properties, including relatively easy access, high proliferative capacity, and the ability to readily differentiate into osteoblasts, BMMSCs are now regarded as the most superior type of MSCs for bone regeneration (20).

Several studies have been conducted to assess the function of BMMSCs in bone tissue engineering. In an *in vitro* study by Zhang et al., BMMSCs were grown in an osteo-induced media after being seeded onto a three-dimensional polycaprolactone/tricalcium phosphate (TCP) scaffold. As indicated by the increased expression of osteogenic genes (RUNX-2, ALP, ON, collagen type I) and calcium deposition, BMMSCs demonstrated an effective osteogenic capacity (21). Hayashi et al., implanted rat BMMSCs/hydroxyapatite composites into a subcutaneous ectopic ossification model, in an *in vivo* study. Six weeks post-implantation, micro-CT imaging and histologic analysis revealed enhanced new bone formation at the surgery site (22). Recent studies have highlighted the significance of biomaterials in modulating the expression of osteogenic genes in BMMSCs and promoting their proliferation and differentiation into osteoblasts. For instance, porous calcium silicate ceramic materials containing silicon and strontium can significantly boost the expression of osteogenic genes in BMMSCs (23). Additionally, the surface characteristics of the magnesium alloy scaffold are essential in boosting the adherence of BMMSCs, along with stimulating their development into bone cells. These attributes also enhance the expression of genes associated with bone formation (24). Furthermore, it has been observed that the osteogenic capacity of BMMSCs varies depending on their origin. Aghaloo et al. (25) found that jawbone-derived MSCs exhibited more osteogenic gene expression and had a higher ability for mineralization compared to long bone-derived MSCs. Similarly, Zhou et al. (26) found that jawbone-derived MSCs exhibited better osteogenic potential and higher levels of Vascular Endothelial Growth Factor (VEGF) secretion compared to femoral BMMSCs under similar culture conditions. According to the *in vitro* investigations by Akintoye et al. (27), iliac crest-derived BMMSCs produced denser bone tissue, while the jawbone-derived MSCs were able to generate more bone tissue under the same induction culture conditions. Collectively, jawbone-derived MSCs can be considered an effective stem cell source for maxillofacial bone regeneration (28).

**FIGURE 2:**
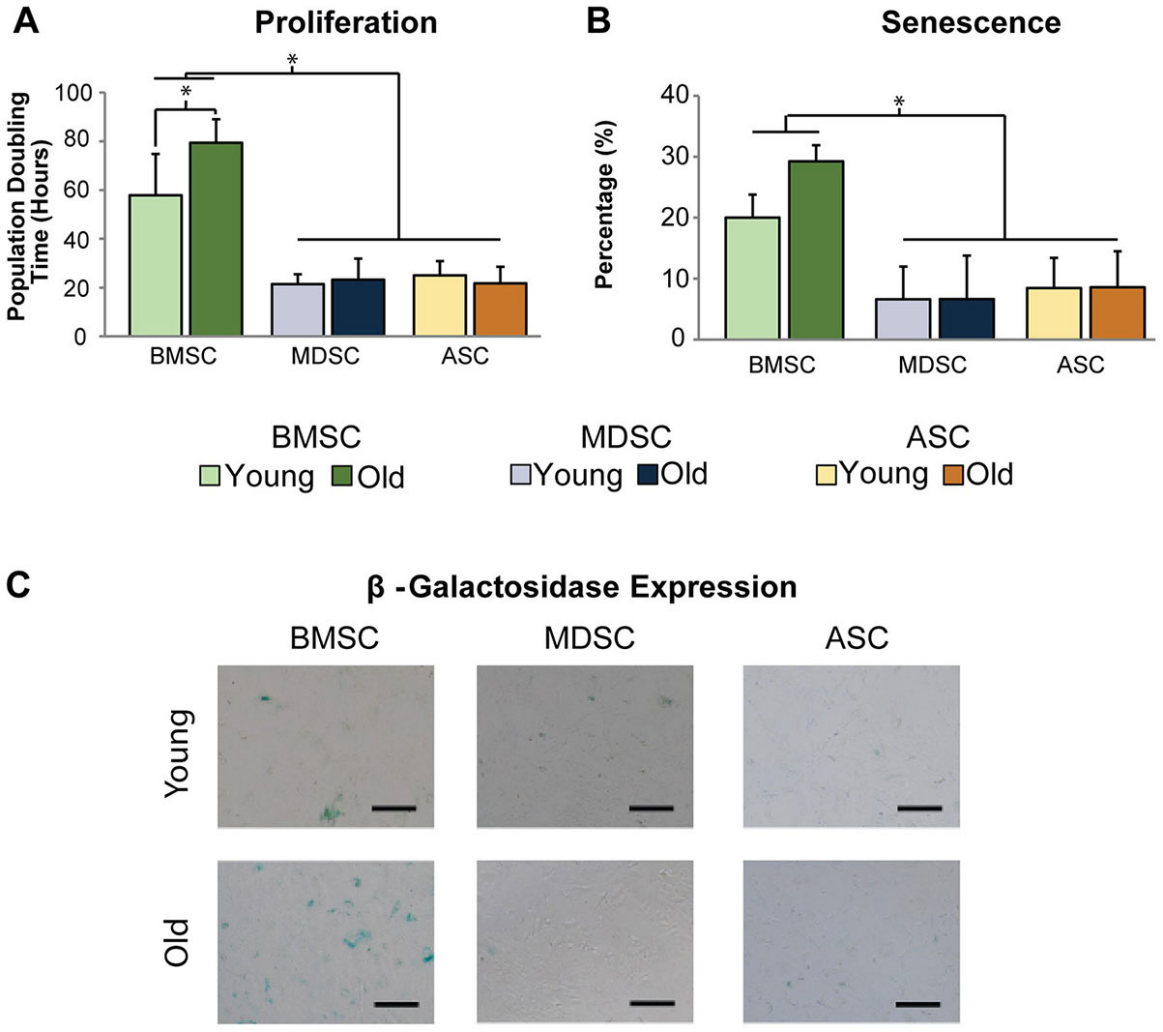
Difference of expansion capacity and senescence of MSCs in age-related changes. **(A)** The population doubling hours (represents the proliferation capacity) of old BMMSCs is obviously longer than those of young BMMSCs. **(B,C)** The quantitative and qualitative research of β-galactosidase staining (represents the senescence) exhibits that the senescence of old BMMSCs is more serious than young BMMSCs, however, the age-related changes of adipose-derived stem cells and muscle derived stem cells are not obvious. [Adapted with permission from Ref. (19)] *p* < 0.05.

Bone marrow mesenchymal stromal cells are usually isolated from anatomical sites as the sternum or pelvic region during bone marrow transplants or orthopedic surgeries and are associated with patient suffering together with potential serious complications (29). Additionally, the available amounts are limited and their senescence increases with patients’ age (Figure 2) (19). These are the main challenges for their application in bone regeneration (Table 1).

#### 2.1.2 Adipose-derived mesenchymal stromal cells

Adipose-derived MSCs (ADSCs) were initially recognized and described as BMMSCs substitutes for bone tissue engineering in 2001 (30). ADSCs can be obtained by liposuction under local anesthesia, commonly from superficial subcutaneous adipose tissue (31). Compared to BMMSCs isolation, their extraction can be painless for patients while providing enough autologous cells for tissue engineering purposes (32). Besides, ADSCs exhibit similar multilineage differentiation capabilities to BMMSCs, including differentiation into skeletal muscle, adipose tissue, tendon, bone, and cartilage. Additionally, the quantity of ADSCs obtainable from adipose tissue and their proliferation capacity are both greater than those of BMMSCs. Furthermore, they can be cultured *in vitro* for extended periods with a low apoptosis rate (33). *In vitro* studies have demonstrated that the osteogenic potential of ADSCs is not diminished with cell proliferation, in contrast to BMMSCs. Moreover, unlike BMMSCs, the osteogenic potential and the expression of osteogenic genes of ADSCs from elderly patients was similar to that of ADSCs isolated from younger patients (34–36).

The clinical use of ADSCs includes implantation into bone defects using undifferentiated ADSCs, ADSC-derived extracellular vesicles (EVs), or ADSC-derived osteoblasts following *in vitro* differentiation. Longaker’s group conducted initial *in vivo* study where they seeded murine ADSCs on apatite-coated poly-(lactic-co-glycolic acid) (PLGA) scaffolds to evaluate their ability to regenerate bone tissue (36). Lendeckel et al. used a combination of autologous ADSCs with fibrin glue to address cranial defects (37). According to experimental evidences, EVs produced by human ADSCs can be progressively and consistently released when immobilized under mild chemical conditions on polydopamine-coated PLGA scaffolds. EVs secretion can enhance the proliferation, osteogenic differentiation, and migration of human MSCs. Furthermore, the results obtained from experiments conducted on living organisms demonstrated that this approach significantly enhanced the process of bone regeneration in a model with a critical-size bone defect (38). Mesimäki et al. presented an innovative approach for addressing maxillary defects in an adult by using the patient’s own ADSCs along with recombinant human bone morphogenetic protein-2 (BMP-2) and β-tricalcium phosphate (β-TCP) granules. After eight months from surgery, the patient showed the growth of new, fully developed, healthy, and well-supplied bone, which successfully fused with the surrounding tissue and remained stable (39). Thesleff et al. employed ADSCs as alternative approach for calvarial reconstruction with successful results in adult patients (40). Several *in vivo* studies showed that ADSCs have a greater ability to promote angiogenesis compared to BMMSCs, especially when cultured under hypoxic conditions, showing a 5-fold increase in VEGF secretion. BMMSCs and ADSCs were placed in PLGA scaffolds and then implanted into a subcutaneous pouch in nude mice. The results highlighted that the number of newly formed blood vessels and bone tissue characteristics were significantly higher/better than that obtained using scaffolds alone or cells alone. This synergistic effect can reduce the number of implanted cells and thus reduce the costs associated with bone regeneration (41–43). Overall, ADSCs can be employed in combination with various scaffolds and growth factors to facilitate bone regeneration in several conditions.

Certainly, heterogenicity of the stromal vascular fraction cells obstacles the effective separation of ADSCs (44). In addition, the process of ADSCs freezing and thawing diminishes their capacity to induce bone regeneration with a significant negative effect on the growth and proliferation of cells *in vitro* (45). Furthermore, ADSCs obtained from patients with systemic diseases like osteoporosis may have a diminished osteogenic potential (46). Several studies have confirmed that the osteogenic capacity of ADSCs is lower than that of BMMSCs. Although both of them showed good proliferation and differentiation when cultured on chitosan/β-1,3-glucan/hydroxyapatite scaffolds, BMMSCs still showed greater adherence, and proliferative capacity (41, 47).

A further source of ADSCs, with potential applications in bone tissue engineering, has been recently identified in infrapatellar fat pad (IFP) adipose tissue. In fact, according to *in vitro*-characterization studies, IFP-derived stem cells show a differentiation potential toward chondrogenic, and osteogenic lineages producing mRNAs of COL1A1, SPARC, and GLUT1; moreover, the significant expression of cortactin gene suggests that their differentiation might be regulated by mechano-transduction. However, despite being an alternative to subcutaneous ADSCs, it must be considered that they might be reprogramed by the inflammatory environment. More investigations are needed for a broad understanding of IFP stem cells’ regenerative potential (Table 1).

#### 2.1.3 Dental-derived mesenchymal stromal cells

Dental-derived mesenchymal stromal cells (DMSCs) are stem cells obtained from several oral tissues including periodontal ligament, dental pulp, apical papilla, dental follicle and also gingiva (48). Laino et al. were the first to successfully isolate stem cells from human oral tissues and demonstrate their capacity for self-renewal and differentiation into various cell types. Following the implantation into mice with weakened immune systems, researchers observed the formation of a lamellar bone structure consisting of osteocytes at the implant site (49). Many *in vivo* and *in vitro* studies have demonstrated the osteogenic capacity of various DMSCs. In contrast to BMMSCs and ADSCs, DMSCs are more readily accessible and their isolation is less invasive; particularly, they can be easily harvested from medical debris, making them a desirable potential source of MSCs for bone tissue engineering purposes (50).

Many researches also highlighted other favorable properties of DMSCs over BMMSCs. For example, the proliferation rate of DMSCs such as dental pulp stem cells, dental follicle stem cells, and the proliferation rate of periodontal stem cells was higher than that of both BMMSCs and ADSCs (51, 52). Although different DMSCs all originate from neural crest cells that arise from the embryonic ectoderm germ layer, there are differences in their phenotypes and osteogenic potential (53, 54). The osteogenic capacity of periodontal stem cells and dental follicle stem cells is only slightly lower than that of BMMSCs and ADSCs, and are both higher than that of dental pulp stem cells and stem cells from human exfoliated deciduous teeth (SHEDs). In a comparative study focusing on SHEDs and dental pulp stem cells, it was demonstrated that SHEDs possess a greater osteogenic potential, while dental pulp stem cells are more likely to produce osseous dentin than bone tissue. This may be attributed to a higher degree of “stemness” and pluripotency in SHEDs (55, 56). Nakajima et al. (57) conducted a comparative study between dental pulp stem cells, SHEDs, and BMMSCs for bone tissue engineering. A scaffold made of PLGA was employed for calvaria defects repair in immunodeficient mice. The results showed that SHEDs are associated with the greatest amount of osteoid and collagen fibers which were also spread extensively. Additionally, the study determined that SHEDs have sufficient bone regeneration capability to effectively restore bone defects. A comparative study was conducted by Vater et al. to assess the bone regeneration capacity of DPSCs and BMMSCs in presence of mineralized collagen matrix (MCM) scaffolds in a critical-size calvarial defect. The results indicated that dental pulp stem cells had a significantly inferior capacity to repair the lesion than BMMSCs (58). Moreover, as showed by Alge et al., the dental pulp stem cells had a greater degree of efficiency in undergoing osteogenic differentiation compared to the BMMSCs (59). A study used hydrogen peroxide and serum deprivation to mimic a potentially harsh microenvironment after transplantation to induce stem cell apoptosis. It was demonstrated that dental pulp stem cells and periodontal stem cells were more resistant to apoptosis than dental follicle stem cells and umbilical cord MSCs which is similar to BMMSCs and ADSCs (29) Taken collectively, periodontal stem cells provide the best alternative to BMMSCs in DMSCs, and SHEDs are also an appealing option for MSCs (Table 1).

### 2.2 Embryonic stem cells

Embryonic stem cells (ESCs) are the cells obtained from the inner cell mass of blastocysts at the pre-implantation stage up to 4 days after fertilization. These cells have a high degree of pluripotency, enabling them to differentiate into any cell type found in the body. Additionally, they have an unlimited capacity for self-renewal and can be guided to transform into osteoblasts when exposed to specific conditions. This renders them highly promising for bone tissue engineering applications (60, 61). In an *in vivo* study, researchers combined human ESCs, stimulated by dexamethasone for 24 h, with sterile poly-D, L-lactide foams and implanted them into Severe Combined Immunodeficient (SCID) mice. After 35 days, mineralized tissue formation was observed at the implantation site without evidence of teratoma. (62). However, ESCs are available only in limited quantities, and their application is ruled by the ethics and laws of many countries (63). Besides, the direct use of ESCs in treating bone defects carries a non-negligible risk of teratoma formation (64). Therefore, exerting a precise control over the differentiation toward osteoblasts and maintaining optimal osteogenic culture conditions *in vitro* are very important (65). Besides, to ensure safety in tissue engineering, ESCs must be correctly committed to the desired lineage at the time of implantation (66). However, if ESCs are differentiated into desired cell types *in vitro* to avoid the risk for teratomas, they can cause an immunological response when transplanted, thus the safety of the treatment cannot be guaranteed (67). These challenges have restricted the utilization of ESCs in bone regeneration (Table 1).

### 2.3 Induced pluripotent stem cells

Induced pluripotent stem cells (IPSCs) are mature differentiated cells that have been reprogrammed to a pluripotent state. This reprogramming is achieved by inducing the expression of four specific transcription factors known as a reprogramming cocktail, which typically includes Oct4/Sox2/c-Myc/KLF4 or Oct4/Sox2/NANOG/LIN28 (68–70). Therefore, IPSCs can be developed as cell lines, as they are programmable to generate multiple cell types from a single cell (71). In contrast to MSCs, which are limited to differentiating into mesodermal tissues, IPSCs can differentiate into ectodermal, mesodermal, and endodermal tissues. This enables a broader spectrum of applications (72).

Presently, recurring to IPSCs is becoming a desirable option for bone tissue engineering overcoming the potential risk of teratoma associated with ESCs (64). Prior research has demonstrated that the ability of IPSCs to form bone tissue is comparable to that of ESCs (73). However, this does not mean that IPSCs will be safer to use than ESCs, some other researches have demonstrated that IPSCs are at a higher risk of tumor development. This risk may be attributed to the re-reprogramming process, which frequently employs genes that have high expression in a variety of cancers (64, 74). In recent years, new induction techniques have been developed to reduce the tumorigenic risk. These protocols avoid the use of oncogenic transcription factors, like c-Myc (70). There are research that also suggest to differentiate IPSCs before implantation. It is crucial to ensure that no undifferentiated cells are introduced during the implantation process to prevent contamination (75). Despite promising, IPSCs suffer from genomic instability and immune rejection, so their application is at a preliminary stage (76).

## 3 MSCs derivatives and bone regeneration

Mesenchymal stromal cells derivatives are divided into two categories: extracellular vesicles (EVs) and bioactive factors. Evs have emerged as pivotal mediators within tissues; certain proteins, lipids, and nucleic acids found in Evs can be transferred and operate as signaling molecules to change cellular behavior (77). Thus, Evs have a key role not only in guaranteeing normal physiological processes but also in regulating several disease-related mechanisms (78–80). Based on their biogenesis, Evs have been classified into three main subgroups: exosomes, microvesicles, and apoptotic bodies (80–82). However, in accordance with the latest consensus guidelines “Minimal Information for Studies of Extracellular Vesicles” (MISEV2023) from the International Society for Extracellular Vesicles (ISEV), this nomenclature is “discouraged” unless the subcellular origin can be proved; conversely, the term Evs is “recommended” (83). As for the “operational terms” like small Evs (diameter < 200 nm) and large Evs (diameter > 200 nm), although their use is allowed, caution is required, as this classification is possibly influenced by the method used for characterization (84).

Bioactive factors encompass a range of interleukins, cytokines, chemokines, proteins, and growth factors, as well as cell-free nucleic acids (such as miRNA, mRNA, and lncRNA), and lipids (such as sphingolipids, cholesterol, and ceramides) (85).

### 3.1 Extracellular vesicles

**FIGURE 3:**
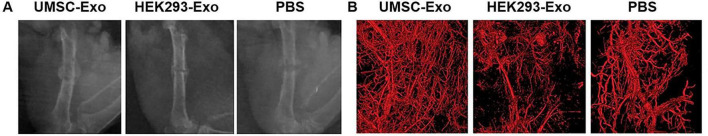
Radiographic analysis of the fracture healing in different conditions. **(A)** Representative X-ray images of the fractures on post-operative day 14, the uMSC-Evs group had larger callus volumes than those of the HEK293-Evs and PBS groups (served as control groups) **(B)** Callus and vessel volumes were reconstructed and qualitatively evaluated by high-resolution micro-CT, the employment of uMSC-Evs led to obviously increased vessel volumes. [Adapted with permission from Ref. (95)].

Extracellular vesicles are membrane vesicles released by most cultured cells that facilitate cell-cell communication through transferring bioactive substances like proteins or nucleic acid to the recipient cells (86). MSCs also release Evs, and studies show that MSC-derived Evs serve a similar function in tissue repair as MSCs (86, 87). The present mainstream view is that the benefits of MSCs in tissue regeneration are attributed to secreted nutritional factors, among which Evs may play a key role (87). Due to the above characteristics, Evs are recognized as appealing molecules in bone tissue engineering, with a significant position in cellular regeneration therapy of bones. The majority of research has demonstrated that Evs regulate bone regeneration-related pathways (e.g., immunomodulatory effects during bone regeneration (87), enhance local angiogenesis (88), microRNA helps regulate the process of bone regeneration (89), and many other aspects). Researchers have proved that Evs produced from MSCs can regulate osteogenic-related pathways, including the Smad pathway activated by Bmpr2/Acvr2b competitive receptors (90), adjusting the TAF15/RUNX2 to transmit SNHG7 (91), blocking excessive activation of the canonical Wnt signaling pathway (82), activating the AKT/mTOR pathway and other pathways involved in bone regeneration (92). The properties of Evs that promote bone regeneration depend critically on the host’s immunological response to them (93). According to the findings, dental pulp stem cell-derived Evs (DPSC-Evs) help switch macrophage phenotype from M1 to M2. Additionally, in rats with experimental periodontitis, Qiao et al. found that DPSC-Evs were able to stimulate periodontal epithelium healing and prevent alveolar bone loss *in vivo* (88). A study has proved that MSC-Evs stimulate early angiogenesis and support bone regeneration (94). The Evs of human umbilical cord MSCs facilitate healing of the bone fracture by promoting angiogenesis with HIF-1α (Figure 3) (95). The miR-21/notch1/dll4 signal pathway may be involved in this process, which enhances angiogenesis to mend massive bone defects (96), the Evs secreted by human deciduous tooth stem cells regulate angiogenesis and osteogenesis through the AMPK signaling pathway, promoting alveolar bone regeneration and facilitating periodontal bone regeneration (97). Endogenous non-coding ribonucleic acid microRNA binds to the 3′ untranslated region (UTR) (or 5′ seed region) of the target messenger ribonucleic acid, hence acting as a negative regulatory factor for post-transcriptional gene expression (98). By transferring miRNA to recipient cells, Evs can control epigenetic processes and the biological function of those cells in bone remodeling. The MSC-Evs are now implicated in the bone regeneration process in a variety of ways. These include miR-1260a (99), miR-31 (100), miR-375 (101), miR-23a, miR-17 (101), miR-182 (101), and many others. Apart from the previously discussed potential processes, researchers have suggested that PLIN5-driven regulation of lipid metabolism is the means by which Evs produced from MSCs regulate bone remodeling (102). It was discovered that BMSC Evs mediate the autophagy level of MC3T3-E1 cells, which stimulates osteogenic differentiation (103). Although the majority of recent research has demonstrated that Evs are essential for bone repair, efforts are still needed to truly apply them in clinical practice, as there are limitations such as insufficient availability of extracellular vesicles and the influence of donor age (104).

### 3.2 Bioactive factors

**FIGURE 4:**
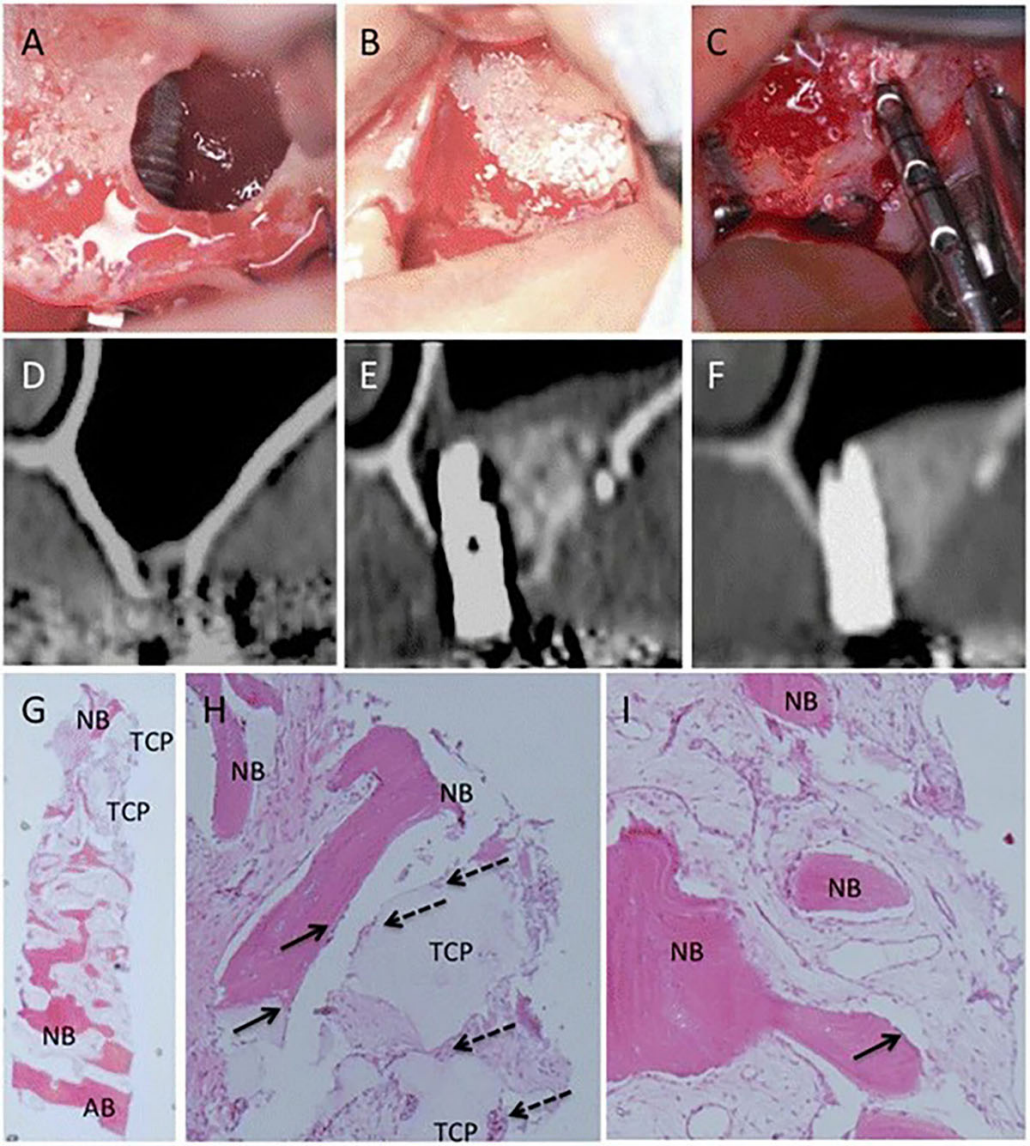
Clinical, radiographic, and histological observations of a woman in maxillary sinus floor elevation (SFE) and simultaneous MSC-CM/β-TCP implantation. **(A,B)** The implant and MSC-CM/β-TCP is implanted into the position of SFE surgery **(C)** The implant position was almost covered with newly formed bone and residue of β-TCP after 6 months, and the osseointegration of the implants is satisfactory. **(D)** CT images before SFE procedure I **(E,F)** 3 months and 6 months after SFE and MSC-CM/β-TCP implantation. **(G–I)** Histologic findings of the newly formed bone 6 months after the operation. The residual β-TCP is replaced from the edge by the new bone (NB) throughout the biopsy sample and infiltration of inflammatory cells was not severe. (G, ×12.5) (H, ×100) (I, ×100) [Adapted with permission from Ref. (84)].

Bioactive factors play important roles in cell proliferation, differentiation, and immune regulation. Growth factors, chemokines, inflammatory cytokines, and other bioactive substances are associated with bone regeneration (105). Similarly, MSCs can secrete bone-regeneration-related bioactive factors, which may be another pathway for MSCs to exert bone regeneration effects. Yamada, et al. used cytokine antibody array and Enzyme-Linked Immunosorbent Assay (ELISA) to detect the cytokine secreted by BMMSCs, DPSCs, and deciduous tooth stem cells. The results highlighted that 11 cytokines were associated with tissue regeneration, including growth factors (e.g., Angiogenin, HGF, TGF-β1, EGFR), chemokines (e.g., MCP-1, MCP-2, GRO), and inflammatory cytokines (e.g., Osteoprotegerin, TIMP-2, IL-6, LAP) and were secreted by the three sources of MSCs (105). Mesenchymal stromal cells conditioned medium (MSCs-CM) contains several bioactive factors. Katagiri et al. (84) conducted the first clinical study on alveolar bone restoration by using human MSCs secretome, in which he found that MSCs-CM is safe to use, is responsible of fewer inflammatory symptoms, and has enormous osteogenic potential in bone tissue engineering (Figure 4) (84). In recent years, an increasing number of scholars have used freeze-dried MSCs-CM for research on bone tissue engineering and found that freeze-dried MSCs-CM and its byproducts can enhance bone cells’ osteogenic potential and the production of new bone (106–108). Some scholars have also begun to study the possible pathways for MSCs-CM to promote bone regeneration. Freeze-dried MSCs-CM contains bioactive factors (chemokines, cytokines, etc.) that stimulate macrophage polarization and adjust the surrounding microenvironment to promote osteogenesis (109). The bioactive components interact to form a network that has osteogenic effects. Consequently, freeze-dried MSCs-CM is a promising derivative of MSCs. Bioactive factors share with EXOs the same critical issues associated with storage.

Both Evs and secreted bioactive factors have important advantages in bone regeneration and thus, potentially also in bone tissue engineering. Additionally, more studies in these fields will help to develop promising tissue restoration options for patients. If future breakthroughs are made in the production and storage of stem cell derivatives, it is these novel resources may be used also in clinical practice.

## 4 Biomaterials for scaffolds in bone tissue engineering

Scaffolds are the third essential component in tissue engineering, together with cells and biological active growth factors. Therefore, the selection of materials for scaffold construction and their specific structural design is of paramount importance (110). Scaffolds provide structural support by forming a three-dimensional (3D) framework that facilitates cell adhesion, development, and proliferation, while also promoting the deposition of ECM. Certainly, in addition to biocompatibility and an appropriate biodegradation rate, which ensures the scaffold is gradually replaced by host tissue, bone scaffolds are expected to show adequate stiffness, pore sizes (> 100 μm), surface topology, load-bearing capacity (111). The exceptional strength of bone, with a tensile strength ranging from 700 to 1400 kg/cm^2^ and compressive strength from 1400 to 2100 kg/cm^2^, is primarily due to the alignment of collagen fibers and mineral crystals parallel to the bone’s long axis. Additionally, the elasticity of bone tissue, with a modulus of elasticity estimated between 420 and 700 kg/cm^2^, is essential for proper bone function (110).

Moreover, bone scaffolds must be manufactured in a specific shape in order to adapt to complex bone defects and trigger osteoinduction, osteoconduction, and osteogenesis. Osteoinduction induces the differentiation of cells by activating external growth factors, which in turn initiates bone formation; osteoconduction, on the other hand, creates the matrix facilitating bone cell adhesion. Besides, the effective bone generation by cells can be induced by osteogenesis (112, 113).

Different biomaterials are used in bone-related applications; briefly, these include polymers, ceramics, biodegradable metals, and composite materials from a combination of polymer and ceramics (114).

### 4.1 Polymers

Polymers for scaffolds fabrication are generally classified into natural polymers and synthetic polymers. Natural polymers can be either protein or polysaccharide based. Proteins have amino acid sequences that can promote cell adhesion by integrin-binding domains; scaffolds based on polysaccharides need to be improved by chemical surface modifications, combination with osteoconductive materials, integrin-binding sequences, or cell adhesion proteins. Unfortunately, they are susceptible to contamination and batch-to-batch variability, as well as inadequate mechanical properties, requiring the adoption of strategies (e.g., modulation of concentration, porosity, polymerization conditions, and addition of inorganic compounds) to improve their strength. Collagen, silk fibroin, alginate, chitosan and hyaluronic acid are the most thoroughly researched natural polymers for bone tissue engineering. (115–117).

Collagen is the predominant protein in mammals. There are about 28 different forms of collagen and types I, II, III, and V are essential components of bone, cartilage, muscle, skin, and tendon. Furthermore, they can be found in fibrillar form that exhibit intricate 3D arrangements in the ECM. Bone tissue mainly consists of type I with a little presence of type V collagen. Collagen structure serves as an anchorage for nanosized hydroxyapatite crystals (118). It descends that collagen is an appealing biomaterial for bone regeneration, especially type I; in fact, it distinguishes for biocompatibility, high porosity, hydrophilicity, low antigenicity, and good resorption (3).

However, as collagen displays low mechanical strength and lacks mineralization, embedding additive organic and inorganic materials is a beneficial solution to overcome this limit also enhancing properties like mineralization capability, cell adhesion, and stem cell differentiation (119). These two major categories can be recognized: collagen/inorganic bioactive materials (bioceramics, β-TCP, hydroxyapatite, and graphene oxide), and collagen/polymers (natural polymers including chitosan, alginate, and silk fibroin; synthetic polymers including PCL, PLGA, and PVA) (120). Modifying or combining collagen with other materials it is possible to obtain many different bone substitutes such as sponges, microfibers or spheres, and hydrogel (121).

Salgado et al. (122) studied the behavior of human bone marrow stromal cells (HBMSC) on collagen/nanohydroxyapatite particles (nanoHA) cryogel where, to enhance the osteogenic induction of the scaffold, the nanoHA were homogeneously dispersed over the pore’s walls of the type I collagen matrix. The presence of nanoHA enhanced cellular attachment and spreading *in vitro*; in particular, scaffolds with higher nanoHA content (e.g., 70%) supported greater cell proliferation compared to those with lower concentrations (e.g., 30%) and promoted a more pronounced osteogenic differentiation, as indicated by increased alkaline phosphatase (ALP) activity and osteocalcin (OCN) expression. *In vivo* studies involving subcutaneous and bone implantation in rodent models demonstrated that collagen-nanoHA cryogels facilitated tissue ingrowth and bone formation.

Annamalai et al. (123) showed the ability of injectable MSC-laden chitosan-collagen microtissues in bridging the cavity of the critical-sized calvarial defect in an animal model of disease. Specifically, these chitosan–collagen biocomposites distinguished for osteoconductivity and also showed to enhance MSCs differentiation as providing a suitable microenvironment for cells in bone repair/integration.

Toosi et al. (124) demonstrated that collagen sponges + PGA fibers characterized by interconnected porous structures show both good biocompatibility and degradability, also improving the initial adhesion, proliferation and osteogenic differentiation activities of BMSCs.

Graphene oxide (GO) can enhance the osteogenic differentiation of MSCs. Liu et al. (125) developed a highly porous aerogel made of GO and type I collagen. Different concentrations of GO were adopted: 0%, 0.05%, 0.1%, and 0.2% w/v). *In vitro* studies related results proved that 0.1% GO-collagen aerogel exhibited the better biomineralization rate and cell compatibility within the cohort. Specifically, rat bone marrow mesenchymal stem cells were used. Moreover, the better outcomes were observed for this group also *in vivo*, in rat cranial defect models. Study evidences suggested 0.1% GO-collagen aerogel as promising biocompatible scaffold for bone regeneration and tissue engineering.

Regarding collagen isolation for fabrication of medical devices, the most frequently used sources are porcine skin and bovine skin/tendons. The extraction process involves the use of chemical solutions such as neutral saline solutions or acidic solutions, along with enzymatic methods by employing pepsin, papain, and collagenase (126).

Silk fibroin originates from silkworms, including the *Bombyx mori* (*B. mori*) species, which is the dominant source of silk worldwide. Raw silk consists of two fibroin fibers that run parallel to one another and are bound together by a coating of sericin on the outside. After the process of degumming is completed to remove sericin from the raw silk, the resultant fibroin fibers have a shiny and smooth appearance. Fibroin fibers have a combination of desirable physiochemical properties along with exceptionally remarkable mechanical characteristics (such as fracture strain, strength, and toughness), making them exhibit qualities that are beyond those of several synthetic or natural fibers, thus undoubtedly becoming one of the most remarkable natural protein fibers (127). It can be integrated with calcium phosphate bioceramics such as hydroxyapatite (Hap), β-TCP, or calcium sulfate, which are commonly used as grafting materials in clinical applications (128). Additionally, the combination leads to the formation of silk fibroin-based composite scaffolds, which enhance the adhesion, proliferation, and differentiation of cells. Silk fibroin-based biomaterials could also be developed into several material forms, such as films, hydrogels, sponges, 3D structures, and nanoparticles (127).

Wang et al. (129) showed that BMSCs harvested from rabbits subjected to ovariectomy and transplanted in alginate gels back into the same rabbits lead to enhanced bone formation and stiffness.

Sartika et al. (130) demonstrated the ability of pure 3D silk fibroin scaffolds, seeded with human adipose-derived mesenchymal stem cells (hASCs), in promoting osteogenic differentiation of the cells *in vitro*. Moreover, at six and twelve weeks postimplantation in rat calvarial defect model, histological staining results revealed that the SF-hASCs scaffold was associated with bone extracellular matrix deposition in the defect regions. Immunohistochemical staining confirmed this evidence; osteoblast-related genes (BMP-2, COL1a1, and OCN) were also expressed, suggesting scaffold ability to achieve effective bone remodeling. Silk fibroin proved to be an effective carrier for stem cells, to be used as an osteoconductive bioscaffold for bone tissue engineering.

Alginate is a biopolymer derived from a variety of brown algae. Firstly, the aqueous alkali solution, generally including NaOH, is employed to initially treat the brown algae. Then, the extract undergoes filtration, and the filtrate is combined with calcium chloride to cause the precipitation of alginate. Subsequently, the conversion of the salt into alginic acid can be achieved by subjecting the alginate salt to diluted hydrochloric acid (HCl) treatment. The sodium alginate soluble in water could be obtained after the purifying process (131). Alginate is a linear polysaccharide that includes homopolymeric units of 1,4-linked (-D-mannuronic acid) and (-L-guluronic acid) in its chemical structure. The mannuronic acid block segments exhibit a straight and flexible structure, but the (1→4) connections to guluronic acid form an obstruction around the carboxyl groups. The glucuronic acid block segments contribute to the folded and rigid structural conformation, which is the main cause of the significant stiffness of the molecular chains. Typically, alginates that have a high concentration of mannuronic acid are often immunogenic. Moreover, in comparison to alginates with a high glucuronic acid content, these alginates are also more effective in stimulating the production of cytokines. Contents in mannuronic and glucuronic acid depend on the source of extraction that, in turn, also influences specific block lengths and thus the properties of the material (126, 132). Alginate is extensively utilized in the biomedical field because of its inherent properties such as simple manipulation and affordability, compatibility with living tissues, exceptional mucosal adhesion, ability to form a gel *in situ*, and resistance to degrade. Alginate can be used to create various 3D scaffold materials and the properties of these materials, including osteogenic differentiation, biocompatibility, mechanical strength, cell adhesion, and proliferation, can be influenced by factors including the composition, molecular weight (generally ranging from 32 to 400 kDa), purity, and concentration. High molecular weight alginate is preferable for hard tissues tissue engineering (131, 133).

Salem et al. (127) developed a composite scaffold integrating calcium titanate nanoparticles into a 3D-printed alginate scaffold seeded with MSCs. The support showed to significantly enhanced bone regeneration in critical-sized calvarial defects; moreover, it was detected an improved bone mineral density, nearly complete defect closure in micro-CT imaging, and enhanced histological outcomes with new bone and marrow cavity. According to gene expression analysis, it was observed an upregulation of osteogenic and angiogenic markers, together with downregulation of osteoclast-related genes.

Alginate/gelatin (Alg-Gel) hydrogels were prepared by Ferjaoui et al. to be associated with MSCs and guide bone tissue formation (128, 134). Specifically, the behavior (attachment, morphology, proliferation, and osteogenic differentiation) of dental pulp stem cell (DPSC) was assessed. Briefly, the cells showed good adhesion to the hydrogels and proliferation. A good osteogenic activity was obtained with hydrogels made of 8% alginate and 12% gelatin. The study showed that stiffness of Alg-Gel gel can guide osteogenesis *in vitro* in absence of DPSCs properties alteration.

Chitosan is a deacetylated form of the natural polymer chitin, which is present in the exoskeletons of crustaceans such as shrimp, crabs, and the walls of fungi. Chitin can be converted to chitosan by partial enzymatic or chemical deacetylation; generally, it is regarded as chitosan, chitin with a deacetylation degree that can vary from 30% to 95%. Both chitin and chitosan belong to a family of linear polysaccharides consisting in glucosamine and N-acetyl-D-glucosamine repeated units linked by covalent b-1-4-glucosidic bonds that are disrupted, especially by enzymatic reactions, under biodegradation (135–137). Chitosan is an alkalescent compound that is not soluble in water or organic solvents. However, it can dissolve in acidic solutions with a pH lower than 6.5 by undergoing protonation of its primary amine groups (138, 139). With a molecular weight (MW) of 1.2 × 10^5^ g/mol, it shares a structural similarity to glycosaminoglycan, an essential element of bone matrix and cell surface (140). This similarity allows it to regulate the availability and function of many osteoclastic and osteogenic factors (141, 142). Chitosan displays keynote characteristics including biodegradability, biocompatibility, anti-infection, antioxidant, and hemostatic properties. Moreover, it can deliver medications or antibiotics in the peri-implant site as a drug carrier (143, 144). Both MW and deacetylation degree modulate chitosan physicochemical properties. Typically, the level of deacetylation is directly related to the solubility, viscosity, biocompatibility, mucosal adhesion, as well as antibacterial and hemostatic activities. Simultaneously, the crystallinity and biodegradability of chitosan diminish when the degree of deacetylation is reduced. Furthermore, the biodegradability and antioxidant activity correspond to the molar mass and tridimensional shape (137). Interestingly, chitosan has been regarded as a superior polysaccharide for fabricating bone scaffolds. However, its mechanical strength is reduced due to its linear form, which hinders its effectiveness in bone tissue synthesis. In consideration of this, recurring to crosslinkers (e.g., dextrins, genipins, and purines) is mandatory (142). Additionally, to overcome this limit, chitosan combination with other materials including natural polymers (collagen, alginate, gelatin, silk fibroin) and synthetic polymers (polylactic acid – PLA, polycaprolactone – PCL, poly-(lactic-co-glycolic) acid – PLGA, poly – l – lactic acid – PLLA), ceramics (calcium phosphate ceramic, bioglass ceramic) has been reported (145).

Zang et al. (146) reported about the development of chitosan-based scaffolds combined with bovine-derived xenografts (BDXs). The chitosan/BDX (mass of 40:60) scaffolds showed significantly enhanced compressive strength than the chitosan scaffold; moreover, they also induced better cell attachment and promoted more osteogenic differentiation of human jawbone marrow-derived mesenchymal stem cells than the CS scaffold. Moreover, for repairing calvarial bone defects. Showed enhanced compressive strength, (hJBMMSCs). The chitosan/BDX composite scaffold with a mass ratio of 40:60 demonstrated superior bone regeneration capacity in critical-size rat calvarial defects, supporting new bone formation and mature lamellar bone formation 8 weeks postimplantation.

Georgopoulou et al. (147) developed crosslinked chitosan/gelatin (CS:Gel, 40:60%) scaffolds with a gel-like, porous structure. These scaffolds supported strong cell adhesion, infiltration, and proliferation of MC3T3-E1 pre-osteoblasts (after 7 days) and human bone marrow-derived mesenchymal stem cells (BM-MSCs) (after 14 days). Compared to standard tissue culture surfaces, the scaffolds significantly increased collagen secretion and enhanced osteogenic gene expression (RUNX2, ALP, OSC) in BM-MSCs. *In vivo* implantation in mouse femurs showed extracellular matrix formation and collagen production by fibroblasts with minimal inflammation, indicating good biocompatibility and osteogenic potential.

Hyaluronic acid (HA) is a simple, anionic, and non-sulfated glycosaminoglycan (GAG) made of repeating D-glucuronic acid and N-acetylglucosamine disaccharide units, linked together by β-1,4 and β-1,3 glycosidic bonds (115, 148). Its molecular weight can vary widely, from a few hundred up to 4 million Da, allowing it to retain large amounts of water due to its hydroxyl groups that are negatively charged (115, 149). Using HA with a molecular weight of 100 kDa falls within an acceptable range in the field of biomedical science (150). Differently from other GAGs, HA is synthesized at the cell membrane by hyaluronan synthases (HAS) enzymes and does not attach to a core protein (151).

Whether diluted in a physiological solution, HA has a gel-like, viscoelastic structure; moreover, it can form a complex macromolecular network displaying a viscoelastic behavior in case the high molar mass of HA and high concentrations are used. However, such viscoelastic material suffer from poor mechanical integrity over the long term. To overcome this limit, HA-based hydrogels characterized by tailored mechanical properties, can be prepared recurring to covalent cross-linking (152). HA is rapidly degraded in tissues by hyaluronidase enzymes, which breaks it into smaller fragments. Due to its biophysical and biochemical properties HA is a key component in biological systems with broad biomedical applications (115).

Chiang et al. (153) demonstrated that intra-articular injection of allogenic MSCs with HA gels in rabbits can prevent osteoarthritis progression better than HA alone. It was observed a reduced formation of osteocyte as well as less subchondral bone exposure and cartilage wearing.

Li et al. (154) showed that BM-MSCs and HA together are effective in improving the femoral trochlear and condyle defects as compared to HA alone in a beagle canine model.

Boekel et al. (155) evaluated the potential of ADSCs combined with HA for bone tissue engineering in rats with critical femoral bone defects. Five treatment groups were compared: control (no graft), HA alone, ADSCs alone, ADSCs + HA, and osteoinduced ADSCs + HA. After 23 days, the ADSCs + HA group showed significantly higher bone contact surface and bone surface density than control and HA-only groups; these results were confirmed by histological analyses. As for gene expression study by RT-PCR, no significant differences were observed in collagen type I or osteopontin levels, but osteonectin expression was elevated in the HA and osteoinduced ADSCs + HA groups. The combination of ADSCs with HA (without prior osteoinduction) improves bone regeneration revelaing effective for bone tissue engineering.

Synthetic polymers, compared to natural polymers, may show lower cell attachment, bioactivity, and osteoconductivity but take advantage of several properties that include tunability, design flexibility, and processability (156). These characteristics allow the fabrication of scaffolds whose characteristics can be modulated according to the specific applications. Additionally, they could be mass-produced and have an extended shelf life compared to natural alternatives (2, 156). Coatings such as bioceramic particles may improve surface performances toward bone regeneration, and aliphatic polyesters, including PCL, PDLA, and PLGA, are the most commonly utilized synthetic polymers (116).

PCL is a biodegradable aliphatic semi-crystalline polymer with a melting temperature above body temperature (about 59°C∼64°C). PCL exhibits a rubbery state characterized by exceptional mechanical qualities at physiological temperature, including high toughness, strength, and elasticity, which vary according to its molecular weight. Along with being non-toxic and biocompatible, PCL also has a longer degradation time (2–3 years) in comparison to other polyester materials. Under physiological circumstances, degradation occurs by microorganisms or by hydrolysis of its aliphatic ester linkage under physiological conditions (2). More specifically, whether used as a biomedical device, PCL experiences a degradation in two stages: first, water is responsible for ester linkages rupture by hydrolytic degradation; then, enzymes perform an intracellular degradation (157). The MW of PCL directly affects the properties of derived scaffolds. Specifically, scaffolds composed of low MW PCL have more hydrophilic and harder surfaces, as well as better mechanical properties in comparison with scaffolds composed of higher MW PCL. These characteristics also contribute to enhanced proliferation and osteogenic differentiation of cells (158). PCL has the potential for load-bearing applications (159).

Xue et al. (160) explored the potential of PCL nanofiber scaffolds in supporting stem cell-based bone regeneration. To this purpose, human MSCs of different origins (umbilical cord, bone marrow and adipose tissue) were cultured on PCL scaffolds. The PCL nanofibers were effective in supporting MSC adhesion, proliferation, and long-term viability. Interestingly, PCL scaffolds significantly enhanced osteogenic differentiation in all MSC types, with bone marrow-derived MSCs showing the strongest effect.

Xu et al. (161) combined 3D-printed polycaprolactone (PCL) scaffolds with BMSCs and self-assembling peptides (SAPs), aiming to enhance both bone regeneration and vascularization. In accordance with *in vitro* evidences, the PCL/SAP scaffolds improved BMSC proliferation and osteogenesis compared to PCL alone. *In vivo* (8-weeks implantation), the PCL/BMSC/SAP scaffolds led to significantly greater bone regeneration and neovascularization than PCL or PCL/BMSC controls.

Polylactic acid is thermoplastic aliphatic polyester that is biodegradable and hydrophobic. Its precursors, which means the lactic acid monomers or lactides, are commonly generated through fermenting renewable agricultural supplies (162). In consideration of the chiral nature of lactic acid, showing two asymmetric centers, it can form three different conformations of isomers (L-PLA, D-PLA, D, L-PLA); thus, different structures with different properties can be prepared. PLA and its isomers have attracted considerable interest in the manufacture of medical implants due to their favorable biological compatibility and mechanical properties. Furthermore, due to gradual degradation and high strength they are suitable for supportive structures and load-bearing constructs since they gradually transfer the load to the adjacent tissue while the damaged part is healing (163).

Bahraminasab et al. (164) considered the *in vivo* healing of critical-sized bone defects in rat calvaria by means of 3D-printed PLA scaffolds, both cell-free and seeded with BMSCs. Histological analysis developed at 8 and 12 weeks post-implantation showed that the two scaffold types were able to significantly enhance healing versus empty controls. Specifically, it was observed the presence of new bone and connective tissue at the defect sites, with the most substantial bone formation and maturation in the stem cell-seeded group at 12 weeks.

Poly-(lactic-co-glycolic) acid is a synthetic linear copolymer and it is possible to obtain various types of PLGA by changing the proportion of lactide acid (LA) to glycolide acid (GA) during the polymerization process (134). As a substitute material for bone, PLGA biodegradability is a fundamental characteristic to consider; in fact, it directly correlates to bone regeneration. Specifically, the LA: GA ratio, monomer order, but also end groups have a role in PLGA devices’ degradation rate, while the MW and the transition temperature value affect its degradation rate (165). The clinical usage of pure PLGA for fabricating bone scaffolds is hindered by its lack of osteoconductivity and inadequate mechanical characteristics in bearing loads. Thus, PLGA is frequently employed with other substances like ceramics or bioactive glass, or it can be properly modified to be more biomimetic and capable of bone tissue engineering (134).

Bhuiyan et al. (166) reported about a novel nano-hydroxyapatite-poly(D,L-lactide-co-glycolide)-collagen biomaterial (nHAP-PLGA-collagen) with mechanical properties similar to that of human cancellous bone. To assess nHAP-PLGA-collagen bone-forming potential, hMSCs were seeded on 2D films and 3D porous scaffolds. Experimental evidence in 2D showed that hMSCs proliferated, formed mineralized nodules, and displayed high ALP activity, suggesting osteogenic differentiation. In 3D scaffolds, hMSCs migrated, filled the porous network, and over 35 days expressed ALP, osteocalcin, and deposited bone-like minerals, without any adipogenic/chondrogenic differentiation. These results highlighted the scaffold selectivity in supporting osteogenesis, revealing it as a promising candidate for bone regeneration.

### 4.2 Bioactive ceramics

Ceramics are formed by applying heat or heat with pressure to a mixture of at least one metal and a non-metallic solid or a non-metal, or a combination of at least two non-metallic solids. They are characterized by great mechanical strength, strong biological compatibility, and minimal biodegradability, which typically makes them unsuitable for tissue engineering applications. However, ceramic or ceramic derivatives are widely utilized in bone regeneration because of their osteoconductive capacity. To address the issue of low biodegradability, introducing porosity with interconnected pores into the derived scaffolds is a key strategy. This porosity not only enhances biodegradability but also stimulates tissue ingrowth. (167). Due to the absence of protein content, there have been no reports of immunological responses, foreign body reactions, or systemic toxicities associated with their use (168).

The calcium salts of orthophosphoric acid, named calcium phosphates, are able to generate compounds that are composed of H_2_PO^4–^, HPO_3_^2–^, or PO_4_^3–^. Tricalcium phosphate (Ca_3_(PO_4_)_2_ and hydroxyapatite Ca_10_(PO_4_)_6_(OH)_2_ are the two main biologically relevant calcium phosphate salts for bone (169). The properties of calcium phosphates have a significant impact on bioactivity, particularly in terms of their ability to promote adhesion, proliferation, and osteogenesis in osteoblasts. To exhibit these bioactive features, degradation and ion release in calcium phosphates are important. These events increase the local concentration of calcium and phosphate ions and stimulate bone minerals formation on calcium phosphates surface (170). Derived biomaterials have gained wide attention by virtue of their excellent biocompatibility, bioactivity, and similarity to bone mineral components (171).

Interestingly, ceramics can be combined with PLA-based polymers to form composites that attract extensive attention for their potential to link the customizable degradability and efficient release properties of polymers with the osteoconductivity and sustained release features of ceramics (167). Certainly, the ideal properties of a ceramic composite are: (i) a certain biodegradation rate to assure bone remodeling; (ii) microporosity to support cell ingrowth; (iii) mechanical stability/ease of handling; (iv) osteoconductivity; (v) growth factors/cells delivery.

Gendviliene et al. (172) evaluated 3D-printed PLA/HA-based scaffolds for bone regeneration using a critical-size calvarial defect model in Wistar rats. The authors compared three groups consisting in: negative and Bio-Oss^®^ controls, PLA and PLA/HA scaffolds, and PLA/HA scaffolds improved with dental pulp stem cells or ECM. After 8 weeks, analyses based on micro-CT and histology highlighted that PLA/HA ECM scaffolds were able to guarantee outcomes in terms of bone regeneration comparable to that of Bio-Oss^®^. Notably, the PLA-only group was associated with marked inflammatory reactions during degradation. Overall, study results suggest PLA/HA ECM scaffolds as promising bone graft alternatives, suggesting further research on ECM effects and material ratios.

### 4.3 Biodegradable metals

During the last 20 years, biodegradable metallic materials have been broadly investigated as promising candidates for the repair of bone tissue because of their ability to degrade naturally over time. Biodegradable metals are materials designed to corrode slowly inside the organism. This corrosion releases products that can be absorbed or processed by cells and tissues. Eventually, the metal completely dissolves, leaving no residue behind (173).

The development of biodegradable metals has focused on iron (Fe), magnesium (Mg), and zinc (Zn), as well as their alloys or composites. Their use in orthopedic surgeries can reduce the issues related to second surgeries for non-degradable metallic implant removal (174, 175).

Fe-based biodegradable materials are highly regarded due to their inner porous structure, exceptional mechanical properties, adaptable shape, biocompatibility, and ability to degrade without releasing hydrogen. In addition, iron and its alloys demonstrate considerable mechanical strength. However, the rate at which they degrade is inadequate to keep up with the bones growth speed (176, 177).

Yang et al. (178) reported about 3D-printed Fe scaffolds with HA nanocoating. The supports showed a precise macropore architecture and a compressive strength within the natural bone range. Overall, scaffold characteristics showed to significantly enhance cell viability, ALP activity, and osteogenic differentiation of rabbit bone marrow stem cells, suggesting 3D-printed, HA-coated Fe scaffolds as promising supports for bone tissue engineering.

Magnesium and its alloys are recognized for their exceptional mechanical characteristics, ability to degrade naturally, and compatibility with living organisms; besides, magnesium alloys have an elastic behavior comparable to that of human bone, making them capable of temporary implants. Within the biological environment, magnesium alloys used for bone replacement could be completely degraded and they can be gradually substituted by newly regenerated tissue without second-surgery requirements. This feature makes them suitable for metallic implants utilized in bone regeneration treatment when the therapy needs transient reinforcement. Unfortunately, devices utilizing magnesium alloys suffer from rapid degradation *in vivo*, resulting in a progressive decline in mechanical capabilities. Additionally, these devices emit harmful by-products because of side reactions and accumulation of corrosion (177).

Lumbikananda et al. (179) investigated the effects of magnesium chloride (MgCl_2_) on the proliferation and osteogenic differentiation of human periodontal ligament stem cells highlighting that low concentrations of MgCl_2_ (from 0.1 to 1 mM) significantly enhanced cell proliferation, colony formation as well as osteogenic differentiation; conversely, higher concentrations (> 10 mM) were cytotoxic. Osteogenic stimulation led to mineralized nodule formation, increased ALP activity, and osteogenic genes upregulation. Overall, 0.1 mM MgCl_2_ was identified as the optimal concentration to support human periodontal ligament stem cells SC function with potential for periodontal and alveolar bone regeneration.

Zinc is an essential microelement of human bodies with the properties of tolerable rates of corrosion and biocompatibility, which makes it appropriate for orthopedic applications. It has been reported to enhance bone repair by promoting cell proliferation, osteogenic differentiation of BMMSCs, formation of vessels, and inhibition of osteoclast differentiation. Moreover, materials with zinc can also play anti-bacterial activity due to bacteria wall damage following reactive oxygen species (ROS) production (180–182). Nevertheless, an excessive amount of zinc in the body can have adverse consequences, such as impairing normal growth and leading to anemia by disrupting iron absorption. Pure zinc has enough mechanical strength in scaffolds. However, pure zinc has some limitations, including lower corrosion rates *in vivo* and relatively inferior mechanical characteristics. Although there are zinc-based biomaterials specifically designed for extended durability to employ in orthopedic surgery, the excessive release of Zn^2+^ during breakdown in the body might have cytotoxic effects and hamper bone integration (177).

Yusa et al. (183) showed that zinc-modified titanium (Zn-Ti) surfaces significantly enhance osteogenic differentiation of human dental pulp stem cells (DPSCs) which showed increased expression of osteoblast-related genes (COL I, BMP2, ALP, Runx2, OPN, and VEGF A), along with higher ALP activity and protein expression. Furtherly, Alizarin Red S staining confirmed enhanced ECM mineralization on Zn-Ti versus controls. Zn-Ti surfaces distinguished for their ability to promote osteogenesis in DPSCs, representing an appealing strategy to promote bone regeneration.

## 5 Bone regeneration by MSCs: an overview on Human Clinical Trials

Current clinical studies recurring to MSCs embedded in scaffolds for bone regeneration were reported in a systematic review by Theodosaki et al., (184) (Table 2). The authors analyzed 14 clinical trials which involved 138 patients suffering from various bone defects which were treated by stem cells combined with scaffolds materials. Different MSCs sources were considered and selected because of their differentiation potential into osteoblasts, ease of harvest, and proximity to the defect site (in accordance with the principle of site-specific tissue repair). These included: bone marrow-derived MSCs (BMMSCs) (from the iliac crest and alveolar bone); dental-origin MSCs such as periodontal ligament stem cells (PDLSCs) and dental pulp stem cells (DPMSCs and DDPSCs); and adipose-derived MSCs, from buccal fat pads (BFSCs) or abdominal tissue (ADMSCs). Preliminarily, all MSCs used were cultured *ex vivo*, expanded to 10^5^–10^7^ cells per graft, and characterized for specific surface markers, in accordance with the International Society for Cellular Therapy (ISCT) guidelines.

**TABLE 2:** Human clinical trials.

Study	Study design	Type of defect	Number of patients	Gender male/female	Age	Type of mesen- chymal stem cell	Type of scaffold	Control group	Inter- vention group	Follow up	Adverse events	Outcomes	Conclusion
Apatzidou et al. (194) NCT02449005 Greece	RCT	Intrabony periodontal defect	27	9/18	20–68	a-BMMSCs	Collagen fleece (Parasorb^®^)	GB: collagen fleece + aFPL GC: MAF	GA: Collagen fleece + aFPL + 5 × 10^6^a-BMMSCs/0.5 cm^3^	6 weeks 3,6,9,12 months 3 years	Not observed	EHI, safety, CAL, PD, recession, radiographic bone fill (BF)	All variables howed significant clinical improvement with no statistical difference between the groups. Greater radiographic improvement in GA-GC/GB
Chen et al. (195) NCT01357785 China	RCT	Intrabony periodontal defect	30 (48intra-bony defects)		18–65	PDLSCs	Bone xenograft BioOss^®^	Graft + GBR	Graft + PDLSCs + GBR	2 weeks, 3,6,12 months	Moderate swelling and pain in some patients	Safety, blood tests, BF CAL, PD, GR	X-ray filling of bone lesions was observed in both groups, with no sta-tistically significant difference between the groups. The increase was pro- portional to time.
Hernandez-Mondaraz et al. (196) ISRCTN12831118 Mexico	RCT	Intrabony periodontal defect	22	14/7	59.4 ± 5.19 (55–64) years	DPSCs	Lyophilized PVP Sponge (clg-PVP^®^)	Collagen sponge + colla- gen membrane (biomed extend)	Collagen sponge + 5 × 10^6^ DPMSCs + collagen membrane (biomed extend)	6 months	Pain con-trolled with painkillers	PD, tooth mobility, bone density (HU), antioxidant and interleukin levels (TAS, SOD, LPO, IL)	The increase in bone density was almost twice as high in the intervention group, with no statistically significant differ- ence between the groups.
Khojasteh et al. (197) NCT02859025 Iran	RCT	Alveolar cleft	10		3 adults, 7 children (8–14 years old)	BFSCs (buccal fat pad MSCs)	NBBM (natural bovine bone mineral) Cerabone^®^	Iliac crest bone graft + collagen membrane	10>^6^ BFSCs + 2 ml NBBM + LRCP/Iliac crest bone + collagen membrane	Every 2 weeks, 6 months	There was a partial dehiscence in one patient and partial exposure of the graft site	Soft tissue healing, volume of bone filling radio- graphically	An increase in newly formed bone was observed in all 3 groups, with the BFSCs + iliac bone group showing the largest increase, with no statistically significant difference
Sanchez et al. (198) EudraCT 2013-00435-77 Japan	Quasi RCT intrabony	Intrabony periodontal defect	20	14/16	25–70 years	PDLSCs	Bone xenograft with collagen BioOSS-Collagen	XBS	1 × 10^7^ PDLSCs + 100 mg XBS	6,12 months	Mild, moderate pain and swelling. Physiological closure of the lesion	PD, CAL, REC, FMPS, FMBS intrasurgically measured size of the lesion, quality of life questionnaire, aesthetic result assessment	An improvement in periodontal markers was observed in all 2 groups, with no statistically significant differ-ence between the groups.
Akhlaghi et al. (199) Iran	CCT	Alveolar bone defect	9	3/6	25,87 years (19,53)	BFSCs	HAM (human amniotic membrane)	Iliac crest bone graft + NBBM + HAM	Iliac crest bone graft + NBBM + HAM + BFSCs	5 months	Not observed	Clinical healing, radiographic deficit filling, the feasibility of placing implants	Greater bone healing was observed vertically and horizontally in the intervention group without statistical significance
Ismail et al. (200) NCT01626625 Indonesia	CCT	Non-union of long bone fractures	10	8/2	7–72 years	BMMSCs	HA (hydroxya- patite)	Iliac crest bone graft	14–18 × 10^6^ BMMSCs + HA	1–12 months	Not observed	Assessment of pain, LEFS + DASH to assess functionality, radiographic healing of fracture with Lane-Sandee, Tiedelman	Faster healing b3 months was observed in the intervention group, at 1 year the differences were assimilated between the groups.
Khojasteh et al. (201) Iran	CCT	Alveolar bone defect	8	5/3	38,91 years	BFPSCs (buccal fat pad MSCs)	FDBA (freeze-dried bone allograft SureOss)	Autologous iliac crest bone graft + FDBA	Autologous iliac crest bone graft + FDBA + 1 × 10^5^ BFPSCs	Every 2 weeks, 5 months	No inflammation of a foreign body was observed	Soft tissue healing, X-ray change in bone width, histological % of new bone	A greater increase in bone thickness was observed in the intervention group radiographically, as well as a greater percentage of new bone histologically.
Šponer et al. (202) EudraCT2012-005599-33 Czech Republic	CCT	Femoral bone defect (hiparthroplasty)	37	15/22	44–76 years	BMMSCs	Tricalcium phosphate (β-TCP Vitoss^®^)	(B9) B -TCP (C9) sponge allograft	(A19) 15 + −4.5 × 10^6^ MSC + β-TCP	6 weeks, 3,6,12 months	Not observed	Hip Harris score to assess pain and function, bone healing according to Gie guidelines	Integration of the graft into the intervention group was observed at 6 months and trabecular bone formation at 12 months. There was a significant statistical difference only between group C to B.
Gjerde et al. (203) NCT02751125 EudraCT2012-003139-50 Norway	Single arm	Alveolar bone defect	11(13), 14 sides	4/7	52–75 years average age is 65	BMMSCs	Calcium phosphate biomaterial BCP (HA 20%, β-TCP 80%)-MBCP + ^®^		20 × 10^6^ MSCs/1 cm^3^ + BCP + regenerative membrane (PTFE)	6 months	Not observed	Radiological bone deficit filling, histomor- phometric factors of bone filling, feasibility of placement and osseointegration of implants	An increase in keratinized tissues was observed. An increase in bone was observed both in thickness and volume. Histologi- cally, integration of BCP granules and formation of new bone were observed. Finally, the stability of the implants (ostell values) was increased.
Gomez-Barrena et al. (204) ORTHO-1 NCT01842477 Spain France Germany Italy	Single arm	Non-union of long bone fractures	28(30)	15/13	39 ± 13 years	BMMSCs	Calcium phosphate biomaterial BCP (HA 20%, β-TCP 80%)-MBCP + ^®^			3,6,12 months, with reporting of adverse reactions	19 mild to moderate adverse reactions not related to the interven- tion were observed	X-ray evaluation of fracture healing, Pain reduction (VAS scale)	Gradual healing of fractures was observed, where in 1 year there was complete healing in 92.8% of patients. Healing was delayed in smokers at 6 and 12 months, and to a small extent in tibia fractures. The sex and time since the initial fracture did not affect healing.
Relondo et al. (205) NCT01389661 EudraCT2010-024246-30 Spain	Single arm	Maxillary cyst	9(11)	2/7	36 ± 14 years	a-BMMSCs	3D BioMax serum autologous cross-linked serum- scaffold matrix			2 weeks, 3–4, 6–8 months	Not observed	Clinical assessment of healing, radiographic increase in bone density (HU)	An increase in bone density was observed in all lesions.
Takedashi et al. (206) UMIN000007698 Japan	Single arm	Intrabony periodontal defect	12	2/10	53,25 ± 9,15 ετών(43–72)	ADSCs	Fibrin gel Beriplast ^®^			3,6,9 months	Transient pain, poolitis and dental sensitivity, delayed wound healing	PD, CAL, BOP, GI alveolar bone growth rate, bone filling rate	There was an improvement in periodontal markers as well as the creation of a new alveolar bone, proportionally increasing over time.
Tanikawa et al. (207) NCT01932164 Brazil	Single arm with historical control	Alveolar cleft	6	3/3	10,16 ετών (8–12)	DPSCs	Hydroxy- apatite + collagen sponge (Bio-Oss collagen^®^)	G1: sponge + rhBMP2 G2: iliac crest bone graft	1 × 10^6^ DPSCs + HA and collagen sponge	1,2,3 weeks, 6,12 months	Not observed	Clinical side effects, duration of hospitalization, radiographic deficit filling, tooth eruption.	Satisfactory bone regeneration and tooth eruption (66.7%) and reduced morbidity compared to the control groups.

RCT, Randomized clinical trial; CCT, Controlled clinical trial (non-randomized clinical trial); 3aFPL, autologous fibrin/platelet lysate; MAF, Minimally invasive flap; EHI, Early healing index; CAL, Clinical attachment level; PD, pocket depth; BF, bone fill; REC, recession; GBR, Guided bone regeneration; FMPS, full-mouth plaque score; FMBS, full-mouth bleeding score; OHIP-14, Oral Health Impact Profile-14; VAS, Visual analog scale; LEFS, Lower extremity functionality scale; DASH, Disabilities of the arms, shoulder and hand score; LRCP, Lateral ramus cortical bone; rhBMP2, recombinant human bone morphogenetic protein-2. [Adapted with permission from Theodosaki et al. (184)].

Regarding the clinical conditions addressed, these included: infrabony periodontal defects, alveolar ridge atrophy, alveolar clefts, cystic bone defects of the maxilla, and non-unions of long bones. Different scaffolds were focused, with different compositions which included both natural materials (i.e., collagen sponges and human amniotic membranes) synthetic biomaterials (β-TCP and HA), and hybrid constructs, some of which incorporated platelet lysates or were pre-treated in osteogenic media before implantation.

In terms of trial design, are reported: randomized controlled trials (RCTs), controlled clinical trials (CCTs) and single-arm studies; the follow-up varies from 3 months to 3 years. Briefly, all the studies lead to successful bone regeneration with significant clinical and radiographic improvements; incidence of adverse events was low, often mild and procedure-related (e.g., transient swelling or pain). Interestingly, the combination of MSCs with scaffolds is associated with outcomes that are comparable or superior to that of traditional bone grafting. Moreover, improved bone density, better soft tissue healing, and eventual improved quality of life or implant integration were observed. However, the authors recognize the early phase of many of these studies, where results suffer from a limited sample sizes and a high methodological heterogeneity, limiting the generalizability of the results. Certainly, there is need for more standardized, large-scale, multicenter clinical trials characterized by rigorous study designs in order to validate the efficacy/safety/cost-effectiveness of MSC-based therapies addressing bone regeneration.

## 6 Conclusions and Perspectives

To date, considering the great impact of bone defects management in clinical practice and the shortcomings of autologous and allogeneic bone grafting therapies, such as such as donor site morbidity, high costs, and limited graft size, there is an urgent need for innovative approaches in bone regeneration (87). In recent years, together with advances in bone biology research and growing knowledge of biomaterials, stem cells have proved their attractive potential in bone tissue engineering. This is owing to their capacity to rapidly proliferate, differentiate into multiple lineages, and their low tendency toward senescence. According to tissue engineering principles, the ideal bone scaffold should possess features comparable to those of autograft bone, such as biological safety, long-term viability, and osteogenic and angiogenic properties, while avoiding the constraints inherent to autografts, such as donor site morbidity, high costs, and size limitations (65). Current research has made considerable progress in exploring the types of seed cells and the therapeutic ideas based on stem cell derivatives. However, challenges persist in achieving consistent vascularization in large bone defects as well as in ensuring adequate mechanical properties that match those of native bone, particularly in load-bearing applications (185). In this context, induced tissue-specific stem cells (iTSCs) have emerged as a promising, safer alternative to iPSCs. These cells, generated via transient expression of OSKM factors (OSKM refers to: Oct4, Sox2, Klf4, and c-Myc) from somatic sources such as ADSCs, exist in an intermediate reprogramming state and, interestingly, they do not form teratomas once transplanted, unlike fully reprogrammed pluripotent stem cells. Notably, human iTSCs (hiTSCs) generated from aged ADSCs by a synthetic self-replicating RNA vector retain the ability to proliferate for multiple passages and differentiate into osteoblasts, while expressing markers consistent with their tissue of origin. Their stable expansion, tissue-specific memory, and multipotent differentiation capacity lead the researchers to recognize the iTSCs as a valuable and practical tool for cell-based bone regeneration therapies (186).

Future research is needed to better understand the processes behind stem cell-mediated bone regeneration and fracture repair, as well as the interactive mechanisms between scaffolding materials and seed cells. At present, there is still a lack of reports about the therapeutic use of stem cells and their derivatives in bone tissue engineering (182). Additionally, to date there is increasing evidence reporting about the risk of immune response following allogeneic MSCs implantation, with consequent reduction of their associated therapeutic potential (187). Despite the initial belief that MSCs are immune privileged, several evidence highlight that allogeneic MSC (allo-MSC) therapies are prone to immune rejection. Once transplanted, allo-MSCs often exhibit poor survival and limited engraftment because of recognition and removal by the host immune system. Moreover, experimental evidence have highlighted that allo-MSCs can shift from an immunosuppressive to an immunogenic state in response to environmental cues including inflammation, with consequent activation of both innate and adaptive immune responses. This includes the involvement of CD8^+^ and CD4^+^ cytotoxic T cells, B cells, natural killer (NK) cells, and particularly macrophages, which are involved into rejection via antibody-dependent mechanisms. Because of the formation of donor-specific antibodies and anti-donor memory responses repeat administrations are difficult, in turn increasing the risk of rapid rejection. Moreover, the tissue from MSCs are isolated can influence their immunogenic profile, with adipose-derived MSCs showing higher likelihood of inducing HLA class I-specific antibodies versus bone marrow-derived cells. Because of these challenges associated with the immune response, the therapeutic effectiveness of allo-MSCs is limited in turn highlighting the need for strategies to mitigate immune rejection in MSC-based therapies (188). Acellular strategies, including EVs derived from stem cells, offer promising cell-free alternatives with lower immunogenic risks and improved storage and handling potential (189, 190). In addition, as carriers of stem cells and growth factors, as well as providers of 3D structure for implants, the material selection and structural design of the scaffolds have been extensively studied. Unfortunately, this process still primarily employs the trial-and-error method to obtain ideal scaffolds, which is time-consuming and costly (182). This highlights the need for standardized and predictive scaffold design strategies that can accelerate development while reducing variability. Ideally, next-generation scaffolds may incorporate responsive or “smart” biomaterials capable of sensing and reacting to mechanical, biochemical, or thermal stimuli, thereby adapting to the *in vivo* environment and improving regenerative efficacy. Advances in material sciences, such as hybrid composites with tunable degradation profiles are also contributing to progress in the field (191). In the future, along with the design and manufacture of more desirable bone tissue engineering implants, research methodologies are expected to make significant advances. One of the notable developments will be the application of artificial intelligence in bone tissue engineering. For instance, researchers can use machine learning to model the optimal morphology of scaffolds and the bioactivity of materials to facilitate scaffold design and optimization (192); computational modeling can also be used to predict the response of cells and tissues to a variety of stimuli, helping researchers to better understand the underlying mechanisms of stem cells in bone regeneration (193). These approaches, combined with bioreactor-based tissue conditioning and immune-informed scaffold design, may significantly improve integration and performance of engineered constructs. Greater attention will be devoted to the practical application of stem cells and their derivatives in the medical treatment of bone defects. To ensure successful clinical translation, future efforts must also address methods standardization, large-animal preclinical validation, and long-term safety. Ultimately, integrating stem cell therapy within the broader context of precision medicine - taking advantage from immune modulation, gene editing technologies, and converging advances in bioengineering - will pave the way for more personalized and effective regenerative therapies (185). Certainly, the ideal solution to bone defects appears to be a combination of a biomaterial scaffold, cell biology approaches, and growth factors, within an optimized mechanical environment (190).
